# Tribute to our reviewers – 2023

**DOI:** 10.1002/deo2.343

**Published:** 2024-03-09

The Editorial Board of DEN Open takes this opportunity to acknowledge with particular gratitude the following reviewers who have provided their valuable time and expertise in the evaluation of manuscripts submitted to DEN Open during the period of January 1, 2023 – December 31, 2023.

Takao Itoi

Editor‐in‐Chief

*DEN Open*

Hirofumi Abe

Yasuhiko Abe

Takemi Akahane

Teppei Akimoto

Taiki Aoyama

Naoki Asano

Reiko Ashida

Hideyuki Chiba

Akira Dobashi

Osamu Dohi

Shinpei Doi

Franz Ludwig Dumoulin

Hiroyuki Endo

Katsuya Endo

Mitsuru Esaki

Nao Fujimori

Ai Fujimoto

Toshio Fujisawa

Tomoki Fujita

Mitsuharu Fukasawa

Norimasa Fukata

Shuichi Fukuda

Kohei Funasaka

Kenichi Goda

Atsushi Goto

Osamu Goto

Tae‐Geun Gweon

Shin Haba

Ryunosuke Hakuta

Natalie Halvorsen

Kazuo Hara

Ryo Harada

Kenji Hashimoto

Minami Hashimoto

Shinichi Hashimoto

Tsuyoshi Hayashi

Yukie Hayashi

Masaaki Higashiyama

Takuto Hikichi

Hiroto Hiraga

Kingo Hirasawa

Takashi Hirose

Takashi Hisabe

Ryota Hokari

Masayasu Horibe

Akira Horiuchi

Yusuke Horiuchi

Kinichi Hotta

Shin Ichihara

Ryoji Ichijima

Toshiro Iizuka

Yuichiro Ikebuchi

Atsushi Imagawa

Masahiko Inamori

Kazuya Inoki

Ken Inoue

Tadahisa Inoue

Fumiaki Ishibashi

Yusuke Ishida

Naoki Ishii

Hirotoshi Ishiwatari

Ken Ito

Nobuhito Ito

Masahiro Itonaga

Masaya Iwamuro

Eisuke Iwasaki

Takuji Iwashita

Keisuke Iwata

Mineo Iwatate

Taro Iwatsubo

Yugo Iwaya

Tomohiro Kadota

Hideki Kamada

Ken Kamata

Takashi Kanesaka

Takeshi Kanno

Yoshihide Kanno

Hiromitsu Kanzaki

Motohiko Kato

Shun Kato

Tsunetaka Kato

Hiroshi Kawachi

Takuji Kawamura

Keisuke Kawasaki

Noboru Kawata

Martin Keuchel

Daisuke Kikuchi

Yoshiaki Kimoto

Toshifumi Kin

Satoshi Kinoshita

Katsuya Kitamura

Yoko Kitamura

Hiroki Kiyohara

Hideki Kobara

Hirofumi Kogure

Tomoyuki Koike

Hitoshi Kondo

Jun Konishi

Shinsuke Koshita

Yuta Koyama

Kazuo Koyanagi

Shinsuke Kumei

Shiko Kuribayashi

Hiroki Kurumi

Ryusaku Kusunoki

Takamichi Kuwahara

Masaki Kuwatani

Louis Lau

Shin Maeda

Mai Makiguchi

Noriaki Manabe

Atsuhiro Masuda

Saburo Matsubara

Kazuyuki Matsumoto

Tomoaki Matsumura

Minoru Matsuura

Noriko Matsuura

Kosuke Minaga

Yohei Minato

Yosuke Minoda

Shuichi Miyamoto

Rintaro Moroi

Douglas Motomura

Shuntaro Mukai

Tsuyoshi Mukai

Takashi Murakami

Yasuaki Nagami

Mitsuru Nagata

Itaru Naitoh

Kazunari Nakahara

Yousuke Nakai

So Nakaji

Keiichiro Nakajo

Jun Nakamura

Masanao Nakamura

Hiroko Nebiki

Daiki Nemoto

Ryota Niikura

Tsutomu Nishida

Jun Nishikawa

Noriko Nishiyama

Toshihiro Nishizawa

Tatsuma Nomura

Kouichi Nonaka

Yohei Ogata

Haruei Ogino

Masayasu Ohmori

Masayuki Ohta

Koushiro Ohtsubo

Kazuo Ohtsuka

Shiro Oka

Yutaka Okagawa

Nozomi Okuno

Toru Okuzono

Teppei Omori

Shunsuke Omoto

Satoshi Osawa

Shozo Osera

Kazuhiro Ota

Koji Otani

Ignasi Puig

Yoshiki Sakaguchi

Taku Sakamoto

Naoki Sasahira

Fumisato Sasaki

Takashi Sasaki

Yu Sasaki

Hiroki Sato

Tatsuya Sato

Ryo Seishima

Masau Sekiguchi

Masahiro Serikawa

Kotaro Shibagaki

Satoki Shichijo

Hisashi Shiga

Minoru Shigekawa

Yuto Shimamura

Yuichi Shimizu

Ryo Shimoda

Satoshi Shinozaki

Hideyuki Shiomi

Yoshii Shunsuke

Koichi Soga

Mitsushige Sugimoto

Shinya Sugimoto

Tetsuya Sumiyoshi

Haruhisa Suzuki

Makoto Suzuki

Sho Suzuki

Masahiro Tajika

Naminatsu Takahara

Toshihiro Takamatsu

Yohei Takeda

Kento Takenaka

Mamoru Takenaka

Ken Takeuchi

Naoto Tamai

Mayo Tanabe

Yuki Tanisaka

Tetsuya Tatsuta

Takeshi Tomoda

Ryosuke Tonozuka

Yosuke Toya

Haruka Toyonaga

Takayoshi Tsuchiya

Yoshiki Tsujii

Koichiro Tsutsumi

Kunihisha Uchita

Junji Umeno

Norimitsu Uza

Yoshiki Wada

Masayoshi Yamada

Daisuke Yamaguchi

Tomohiro Yamaguchi

Akira Yamamiya

Kenjiro Yamamoto

Shunsuke Yamamoto

Yasushi Yamasaki

Tatsuhiro Yamazaki

Kei Yane

Takeshi Yasuda

Yoshihiro Yokoyama

Masao Yoshida

Naohisa Yoshida

Daisuke Yoshimura

Shigetaka Yoshinaga

Toshiyuki Yoshio

Tetsuya Yoshizaki

Ryo Yuge

